# The Recent Epidemic of Cholera at the County Hospital

**Published:** 1866-10

**Authors:** T. Bevan

**Affiliations:** Attending Physician to the Hospital


*The Recent Epidemic of Cholera at the County Hospital.* Chicago, 1866. Reported by Dr. T. BEVAN, Attending Physician to the Hospital.

Through the spring and summer months of the current year, until August 6th, our wards at the Hospital contained the usual groups of non-epidemic diseases common in a pauper hospital. The house records show the prevalent phlegmasiæ and acute attacks of other maladies, with a small per cent. of chronic and cachectic cases. No apparent tendency to intestinal troubles beyond an occasional dysentery, or some returned soldier with chronic diarrhœa, had occurred in the Institution to the date above noted, when a Mormon train, said to have some sick among them, abandoned a man named Christian Hansen, at the railroad depot. About noon of the 6th he was admitted to the Hospital, with the following history: He reports himself a native of Denmark, 41 years of age, sick for several days with diarrhœa. He is at present rational, and presents the following symptoms: Extremities very cold, clammy perspiration about the chest and face; capillary circulation very much impaired, giving the surface a dusky hue, especially marked on the lips, about the eyes, and on the hands and fingers; lividity of the roots of the nails; eyes sunk back into their sockets, with the eyeballs turned up, giving the countenance a ghastly appearance; pulse extinct in the radial, and barely perceptible in the brachial artery, quick and feeble, (160 per minute;) absence of apex shock, and cardiac sounds feeble; respiration, 30 per minute, stridulous; tongue swollen and retracted; vomits frequently a thin, glairy serum, slightly tinged yellowish, and only three or four ounces at a time. The discharges from the bowels are involuntary, frequent and copious, of a serous character, mixed with whitish shreds, giving it the genuine "rice-water" appearance; cramps in the abdominal muscles and limbs; considerable tenderness over the abdomen; ankle joints firmly extended, not allowing flexion of the foot upon the leg; urine suppressed; great thirst, continually begging for iced water; shriveling of the integument, especially of the hands, feet and forearms; voice feeble and husky.

*Treatment.*—Bismuth. sub-nit. grs. vi., hydr. sub-mur. gr. i., every half hour until four powders are taken, then every hour. By injection, brandy, ℥i., carb. ammon. grs. v., every hour. Cataplasms of mustard to the spine and abdomen, with general artificial heat to the surface of the body. The man lived five hours, but exhibited no signs of reaction, presenting at last the most excellently marked cholera cyanosis everywhere on the surface.

This report is not made either in the interest of Messieurs, the contagionists, nor their antagonists, the non-contagionists. The subscriber, being a subject for conversion by credible evidence from either party that shall furnish satisfactory proofs, has to say this, however, as a truthful relation of patent facts, that, until noon of August 6th, no case of Asiatic cholera had been reported in Cook County during the year 1866. The case here reported was seen, besides myself, by two other members of the Hospital staff—Dr. R. G. Bogue, the Surgeon on duty, and my colleague, Dr. J. P. Ross, one of the medical attendants—as also by Dr. N. T. Quales, Resident Physician, and his assistants. The patient, Hansen, died between 4 and 5 o'clock the day of his admission, and the cholera question was laid on the table until—

Case II. Mrs. Dawson, aged 25 years, employed as nurse in the Hospital, who had been more or less subject to diarrhœa during the summer, was attacked with unaccustomed severity in the forenoon of August 9th. She had a number of discharges during the morning, but feeling no pain, she gave but little attention to it. Towards noon the dejections recurred at shorter intervals, and she experienced considerable prostration. About 2 P. M., vomiting and cramps set in with great violence, with copious rice-water discharges from the bowels recurring every fifteen or twenty minutes. The surface of the body became cold; the capillary circulation was sluggish, giving the dusky color, so characteristic of the disease, to the skin of the face and extremities. Between the cramping paroxysms and vomiting, the voice was weak but cheerful; respiration, 35 per minute, and somewhat labored; pulse, 150 per minute, quick and feeble. The cramps were principally confined to the legs and feet, though they existed occasionally in the abdominal muscles. There was constant jactitation.

*Treatment.*—Bismuth. sub-nit. grs. iii., hydr. sub-mur. gr. i., morph. sulph. gr. $\frac{1}{4}$, every half hour, or, if the vomiting persisted, immediately after every ejection. Following each discharge from the bowels, give, as an injection, spts. vin. gall. ℥i. + tr. kino, ʒi. + tr. capsici, gtt. iij. Mustard sinapisms to the spine and abdomen. Jugs of hot water, wrapped in blankets, were placed about the body and limbs. This treatment was continued for some time, without any change or abatement of the symptoms. The cramps being very troublesome, morph. hydrochlor. gr. $\frac{1}{3}$, was thrown into the right leg by hypodermic injection. Almost immediately the cramps were moderated; in half an hour they ceased entirely, and did not return for three hours. The vomiting, also, during this time, was less copious, frequent and violent. The amount of alvine discharge seemed also less, but the dejections were involuntary. The first hypodermic injection was given at half-past 3 o'clock P. M. At 8 o'clock P. M., the cramps were again quite violent. The vomiting, also, was more frequent, though the quantity ejected was small and the matter greenish in color. The discharges from the bowels continued to be involuntary, copious, and without any change in color or consistency. [The patient was of a plethoric lymphatic temperament, and lost altogether an enormous quantity of serum.] Another hypodermic injection of morph. sulph. gr. $\frac{1}{4}$, was administered at 8.30 P. M., and the effect was similar to that of the first. At 10 o'clock P. M., the vomiting had ceased; the evacuations from the bowels were less frequent; pulse, 160, weak; respiration difficult; surface of the body cold, though she *complained of heat* and great thirst; the capillary circulation was very much impeded; the voice was husky and feeble; the patient was perfectly indifferent about her condition. I ordered bismuth. grs. vi., hydg. sub-mur. gr. $\frac{1}{2}$, every hour. Capsicum to be added to the injection per rectum, and to be given once each hour, instead of after every discharge. She remained quiet until about 1 o'clock A. M. of the 10th, when the cramps returned with considerable severity. Again they were controlled by hypodermic injection of morph. sulph. gr. $\frac{1}{4}$. From 1 to 2 A. M. there was no vomiting nor purging from the bowels. Between 2 and 3 A. M. there were two small discharges. From 3 to 4 A. M., only one. From 4 to 7 A. M. she remained quiet, and had no injections. Her arms were now very cold, notwithstanding artificial heat and friction were being continually used. Between 7 and 8 A. M. she had one passage from the bowels of a greenish color; the cramps also returned, but were again controlled by hypodermic injection of morph. sulph. gr. $\frac{1}{6}$. About 8 o'clock there appeared to be a feeble attempt at reaction. She was now ordered brandy, ℥i. and gr. v. ammon. carb. every half hour, but the vital spark was too nearly out—reaction was not established. An injection of brandy and ammonia was administered, but without appreciable effect. She now sank rapidly. The pulse, which had been feebly distinguished at the wrist, became again imperceptible, faint, and 200 per minute in the brachial artery. She passed into coma with respirations 10 per minute, gradually growing less frequent. Death occurred at 11.10 A. M., August 10th. No *post mortem* examination.

Case III. William Schnastz, aged 45, native of Germany, had been in the Hospital since January 23d, under treatment for lupus, which had improved very much; ulceration less and general health good; bowels slightly constipated; — was attacked, August 9th, with diarrhœa, for which he was ordered plumbi acet. gr. iii., pulv. opii, gr. i., every four hours. August

10th, he was very much prostrated; during the night he had been on the vessel a number of times, and in the latter part of the night, vomiting, retching, with cramps in the abdomen and limbs. Discharges from the bowels now were more frequent and copious; rice-water stools; pulse extinct at the wrist; respiration labored.

*Treatment.*—Bismuth. sub-nit. grs. iv., hydg. sub-mur. gr. i., every half hour. Injection, per rectum, of brandy, ℥i., tinc. kino, ʒi., tinc. capsici. gtt. iv., after each discharge. Hypodermic injection of one-third gr. of morphine, often enough to control the cramps. August 11th, pulse perceptible in the radial artery, feeble, 130 per minute; respiration continues laborious; discharges from the bowels less frequent, but retaining their light color; vomits frequently; was ordered the following mixture: Acid. sulph. dil. ʒiii., tr. cardam. co. ʒii., aquæ, ℥iijss. M. Dose, a tablespoonful after every discharge. August 12–14, considerable vomiting and diarrhœa still continue, but the patient passes a healthy-looking urine in usual quantity. Ordered the following: Tincts. capsici, camph. and opii, aa. ʒii. M. Dose, gtt. xx. every three hours. August 16th, persistent vomiting and diarrhœa. Ordered a blister, $4 \times 6$, over epigastrium, and to take internally: Tannate of quinine, grs. ii., bismuth. sub-nit. grs. iv., every three hours. This controlled the vomiting and diarrhœa. The old cicatrices of the lupus are all ulcerating anew; appetite and general condition gradually improving.

Case IV. Frederick Ibson, aged 27, native of Denmark, admitted into the Hospital, August 6th, with dysentery, from which he was about recovered, occupying a bed next the one in which Case I. died, was attacked, August 10th, in the forenoon, with vomiting and cramps in the limbs and abdominal muscles, copious watery, light-colored discharges from the bowels. Intense thirst.

*Treatment.*—Bismuth. sub-nit. grs. iv., hydg. sub-mur. gr. i., morph. sulph. gr. $\frac{1}{6}$, every half hour. Brandy injection per rectum. This, with other treatment in the same general direction as the cases previously detailed, was continued; notwithstanding which, the patient sank into collapse and expired, August 12th, at 7 o'clock A. M.

CASE V. Rosalie Reed, aged 28, native of Ireland, convalescing from parturition. During pregnancy, had suffered from albumenuria and general anasarca; after parturition, with ascites. Was attacked, August 11th, with vomiting and frequent diarrhœal discharges of a rice-water character, cramps in the limbs and abdominal muscles.

*Treatment.*—Hydg. sub-mur. grs. iii., piperin, grs. xii., morph. sulph. gr. i. Make into six powders, and give one every two hours. Hypodermic injection of morphine to control the cramps. Injection, per rectum, brandy, ℥i., ammon. carb. grs. v., every hour. August 12th, diarrhœa has ceased; vomiting continues; pulse, 140, feeble. Give bismuth. sub-nit. grs. vi., hydg. sub-mur. gr. $\frac{1}{2}$, every hour; brandy, ℥ss. by injection; beef-tea. August 13th, convalescing.

CASE VI. Mary King, aged 50, native of Ireland, employed as washerwoman in the Hospital, taken, August 12th, with vomiting, cramp, and copious rice-water discharges from the bowels.

Treatment similar to Case V. Recovered.

CASE VII. Miss C——, aged 17, American, a member of the Warden's family, residing in the Hospital, was attacked, August 13th, with diarrhœa, for which she was ordered: Piperin, grs. ii., morph. sulph. gr. $\frac{1}{6}$, hydg. sub-mur. gr. $\frac{1}{2}$, every two hours. This, by noon, had nearly controlled the discharges from the bowels. About 2 P. M., however, cramps and vomiting set in, and soon became something *terrific*—utterly beyond description. It seemed as if every part of the organism capable of contraction was the subject of spasm, including the diaphragm and glottis, interrupting respiration, at times, from ten to fifteen seconds, and ending with a slow whistling inspiration of almost equal duration.

*Treatment.*—One-third of a grain of morphine was exhibited without the slightest effect. Temporary relief was obtained from the inhalation of chloroform, but this was very transient. About 7.30 P. M., the following was given by subcutaneous injection: Atropine sulph. gr. $\frac{1}{30}$, morph. sulph. gr. $\frac{1}{30}$, aq. distill.

gtt. x.—M. This was thrown under the skin of the leg. In twenty-five or thirty minutes the spasm had subsided, except in the glottis and diaphragm, which gradually relaxed, so that in a couple of hours the patient was comparatively relieved. The circulation for three hours after the injection was very irregular. At times, the pulse would be down to 60 per minute; at others, up to 200. Respiration stridulous, and at times slightly stertorous; capillary circulation impeded, giving blue color to the face and extremities. The temperature of the body was considerably increased. At 11.30 P. M., the circulation was more regular; the temperature was better; the dusky hue had subsided; respiration, also, was more regular. At 3 A. M., pulse 135 per minute; respiration, 30 per minute, regular; temperature about normal; patient rational, voice weak and hollow. She was now ordered bismuth. sub-nit. grs. iii., hydg. sub-mur. gr. i., every two hours. About 4 o'clock there was slight cramping of the limbs, and she took internally gr. $\frac{1}{4}$ of morphine, which gave only partial relief. At 6 o'clock the cramps were again quite violent. A foot-bath of mustard, as hot as could be borne, was now ordered. This relieved the spasm almost instantly. It was continued for twenty minutes, when the limbs were dried and wrapped in hot flannels. The patient seemed quite comfortable. We now attempted to administer small quantities of beef-tea, but it was rejected by the stomach. She retained, however, a little milk-punch. During the forenoon she complained of pain in the gluteal muscle of the left side, which was at first attributed to soreness from cramp, but continued examination established the existence of a large carbuncle; subsequently, others were developed on the scalp, shoulder, hip and heel. Poultices of flaxseed meal were applied to all excepting the one on the scalp, to which glycerine and morphine were applied. Towards evening the cramps returned with some violence, and the $\frac{1}{50}$ of a grain of atropine, with $\frac{1}{4}$ of a grain of morphine, were injected under the skin. This controlled the cramps very satisfactorily without the least unpleasant sensation on the part of the patient. She slept comfortably through the most of the night, awaking the following morning free from cramps, of which no renewal occurred. She had a semi-fluid evacuation of whitish color, as if composed of decolorized fæces. August 15th, she was ordered sulphite sodæ grs. x., three times a day, with appropriate nourishment. The carbuncles all subsided, excepting the one on the hip, to which the following was applied: Plumbi iodid. ʒss., ext. opii, aquos, grs. xv., cerat. simp. ℥i., spread thick on lint, and apply under a warm poultice. This relieved the pain and effected resolution, so that from this time the patient rapidly convalesced, and is now (September 4th) quite well.

Case VIII. Sarah M——, aged 26, native of U. S., employed as nurse, in good health, of robust constitution, was attacked at nearly the same time and in like manner, and was similarly treated, as Case VII. The two cases were almost parallel, only in this last the cramps were more confined to the muscles of the back and limbs; there were no carbuncles, and the convalescence was more rapid.

Cases IX, X and XI. Peter Ewander, aged 50, native of Sweden, under treatment for varicose ulcer of the leg; Lewis Larson, aged 28, native of Denmark, convalescing from typhoid fever; and John Lee, aged 35, native of Germany, under treatment for primary syphilis,—were taken, August 13th, with cramp, diarrhœa and vomiting. Treatment similar to Cases V and VI. They all recovered.

Cases XII, XIII and XIV. Mary Carlisle, aged 26, native of Ireland, convalescing from parturition; Ann Good, aged 28, native of England, awaiting delivery in the lying-in ward; and Ann Layton, aged 30, native of Ireland,—were attacked, August 14th, with cramp, vomiting and diarrhœa; evacuations rice-watery, copious and frequent. Treatment similar to Cases V and VI. Recovered.

Case XV. G. C. Town, aged 32, American, was under treatment for typhoid fever, of moderate degree of intensity. August 14th, he was taken with copious rice-water diarrhœa discharges every fifteen minutes, soon becoming involuntary, and in a short time cramps and vomiting set in.

*Treatment.*—Piperin, grs. ii., morph. sulph. gr. $\frac{1}{8}$, hydg. submur. gr. $\frac{1}{2}$, every two hours; brandy and ammon. carb. per rectum after every discharge; artificial heat, mustard sinapisms, etc. In the evening he fell into collapse, and died the following day.

CASE XVI. A. C. Jensen, aged 28, native of Denmark, admitted, August 14th, with diarrhœa. August 15th, was taken with cramps and vomiting, discharges from bowels soon becoming frequent and rice-watery. Towards the evening he sank into collapse, and remained so until August 16th, at 5 o'clock P. M., when he expired. Treatment similar to Case XV. After collapse was established, strychnia and electricity were used freely, the former without effect. The latter, when applied along the spinal column continuously, would, after a time, establish momentarily, but perceptibly, a feeble pulsation in the radial artery at the wrist.

CASE XVII. A woman, about 35 years of age, admitted, August 16th, and was, at the time of admission, in a state of collapse, rice-water discharges, cramp in legs, stridulous respiration, pulse at the wrist extinct, skin cyanosed.

*Treatment.*—Stimulants, artificial heat, mustard sinapisms. No reaction. Died, August 17th.

CASE XVIII. Mary Rhodes, aged 23, American, had been in the Hospital some time with a tumor, supposed to be ovarian, was attacked, August 16th, with diarrhœa, cramp in the lower limbs, and, after a time, with violent vomiting.

*Treatment.*—Bismuth. sub-nit. grs. v., hydg. sub-mur. gr. i., every time she vomits. Hypodermic injections of atropine and morphine in the leg, which controlled the cramps without their return. The vomiting and diarrhœa continuing, brandy and tincture of kino were given by the rectum, with tr. capsici, after each discharge. She also continued the bismuth and calomel. In the evening she sank into collapse, and remained so until August 18th, when she expired at 6 o'clock P. M.

*Post mortem.*—The abdominal tumor was constituted by two ovarian abscesses, one on each side, the right being the largest, containing about twenty ounces of pus; the left was smaller, and about fourteen ounces. The tumors were sacculated, and the cavity of the uterus was found occluded and containing several ounces of pus.

CASE XIX. The last occurring in the house up to date. Samuel White, aged 26, admitted, August 17th, with dysentery, was attacked on the 18th with all the symptoms of Asiatic cholera, and died on the 20th in comatose collapse, after a treatment similar to that adopted in Case XVII.

From that time to the date of this communication no new cases of cholera have occurred in the Hospital. We have the usual diarrhœas and dysenteries of the season, but they manifest no disposition to become fatal or even serious.

*Remarks.*—In addition to the above cases, the Warden of the Hospital and the Resident Physician had distinctly marked choleraic attacks, but recovered. The precautions taken, and the prophylaxis employed, were isolation of the patients, disinfection of the discharges, cleanliness and free ventilation. The epidemic lasted fourteen days. The mortality among the paupers attacked was as 1 to 2, or 50 per cent.; while the mortality among the nurses and officials of the Hospital was as 1 to 5, or 20 per cent. So true is it that pestilential diseases ravage the poor, ill-fed and ill-clothed wrecks of humanity which find their way to a public hospital, that, but for the striking difference noted above, the fact would scarcely need mention; and here let me note the fact, that the cases which occurred among the better class of patients were as violent, if not *more violent*, than among the paupers, yet the former would react and convalesce, while the latter quickly succumbed.

September 15, 1866.

